# Supplementary material for: Prioritising Risk Factors for Prescription Drug Overdose among Older Adults in South Korea: A Multi-Method Study
Source: Int J Environ Res Public Health. 2021 Jun 1;18(11):5948. doi: 10.3390/ijerph18115948 (PMC8198076; doi:10.3390/ijerph18115948)
Supplement: Supplementary file 1 [file ijerph-18-05948-s001.zip › ijerph-1192239-supplementary.pdf]

Table S1. ICD-10 code for prescription drug overdose.

ICD-10 code	Diagnosis
T36	Poisoning by systemic antibiotics
T36.0	Penicillins
T36.1	Cephalosporins and other β-lactam antibiotics
T36.2	Chloramphenicol group
T36.3	Macrolide
T36.4	Tetracycline
T36.5	Aminoglycoside
T36.6	Rifamycin
T36.7	Antifungal antibiotics, systemically used
T36.8	Other systemic antibiotics
T36.9	Systemic antibiotic, unspecified
T37	Poisoning by other systemic anti-infectives and antiparasitics
T37.0	Sulfonamide
T37.1	Antimycobacterial drugs
T37.2	Antimalarials and drugs acting on other blood protozoa
T37.3	Other antiprotozoal drugs
T37.4	Anthelmintics
T37.5	Antiviral drugs
T37.8	Other specified systemic anti-infectives and antiparasitics
T37.9	Systemic anti-infective and antiparasitic, unspecified
T38	Poisoning by hormones and their synthetic substitutes and antagonists, NEC
T38.0	Glucocorticoids and synthetic analogues
T38.1	Thyroid hormones and substitutes
T38.2	Antithyroid drugs
T38.3	Insulin and oral hypoglycaemic [antidiabetic] drugs
T38.4	Oral contraceptives
T38.5	Other estrogens and progestogens
T38.6	Antigonado- trophins, antiestrogens, antiandrogens, NEC
T38.7	Androgens and anabolic congeners
T38.8	Other and unspecified hormonesand their synthetic substitutes

T38.9	Other and unspecified hormone antagonists
T39	Poisoning by nonopioid analgesics, antipyretics and antirheumatics
T39.0	Salicylate
T39.1	4-Aminophenol derivatives
T39.2	Pyrazolone derivatives
T39.3	Other nonsteroidal anti-inflammatory drugs [NSAID]
T39.4	Antirheumatics, NEC
T39.8	Other nonopioid analgesics, antipyretics, NEC
T39.9	Nonopioid analgesics, antipyretics and antirheumatic, unspecified
T40	Poisoning by narcotics and psychodysleptics [hallucinogens]
T40.0	Opium
T40.1	Heroin
T40.2	Other opioids
T40.3	Methadone
T40.4	Other synthetic narcotics
T40.5	Cocaine
T40.6	Other and unspecified narcotics
T40.7	Cannabis (derivatives)
T40.8	Lysergide [LSD]
T40.9	Other and unspecified psychodysleptics [hallucinogens]
T41	Poisoning by anaesthetics and therapeutic gases
T41.0	Inhaled anaesthetics
T41.1	Intravenous anaesthetics
T41.2	Other and unspecified general anaesthetics
T41.3	Local anaesthetics
T41.4	Anaesthetic, unspecified
T41.5	Therapeutic gases
T42	Poisoning by antiepileptic, sedative-hypnotic and antiparkinsonism drugs
T42.0	Hydantoin derivatives
T42.1	Iminostilbenes
T42.2	Succinimide and oxazolidinedione
T42.3	Barbiturate
T42.4	Benzodiazepine

T42.5	Mixed antiepileptics, NEC
T42.6	Other antiepileptic and sedative-hypnotic drugs
T42.7	Antiepileptic and sedative-hypnotis drugs, unspecified
T42.8	Antiparkinsonism drugs and other central muscle-tone depressants
T43	Poisoning by psychotropic drugs, NEC
T43.0	Tricyclic and tetracyclic antidepressants
T43.1	Monoamine-oxidase-inhibitor antidepressants
T43.2	Other and unspecified antidepressants
T43.3	Phenothiazine antipsychotics and neuroleptics
T43.4	Butyrophenone and thiothixene neuroleptics
T43.5	Other and unspecified antipsychotics and neuroleptics
T43.6	Psychostimulants with abuse potential
T43.8	Other psychotropic drugs, NEC
T43.9	Psychotropic drug, unspecified
T44	Poisoning by drugs primarily affecting the autonomic nervous system
T44.0	Anticholinesterase agents
T44.1	Other parasympathomimetics [cholinergics]
T44.2	Ganglionic blocking drugs, NEC
T44.3	Other parasympatholytics [anticholinergics and antimuscarinics] and spasmolytics, NEC
T44.4	Predominantly α-adrenoreceptor agonists, NEC
T44.5	Predominantly β-adrenoreceptor agonists, NEC
T44.6	α-Adrenoreceptor antagonists, NEC
T44.7	β-Adrenoreceptor antagonists, NEC
T44.8	Centrally acting and adrenergic-neuron-blocking agents, NEC
T44.9	Other and unspecified drugs primarily affecting the autonomic nervous system
T45	Poisoning by primarily systemic and haematological agents, NEC
T45.0	Antiallergic and antiemetic drugs
T45.1	Antineoplastic and immunosuppressive drugs
T45.2	Vitamins, NEC
T45.3	Enzymes, NEC
T45.4	Iron and its compounds
T45.5	Anticoagulants
T45.6	Fibrinolysis-affecting drugs

T45.7	Anticoagulant antagonists, vitamin K and other coagulants
T45.8	Other primarily systemic and haematological agents
T45.9	Primarily systemic and haematological agent, unspecified
T46	Poisoning by agents primarily affecting the cardiovascular system
T46.0	Cardiac-stimulant glycosides and drugs of similar action
T46.1	Calcium-channel blockers
T46.2	Other antidysrhythmic drugs, NEC
T46.3	Coronary vasodilators, NEC
T46.4	Angiotensin-converting-enzyme inhibitors
T46.5	Other antihypertensive drugs, NEC
T46.6	Antihyperlipidaemic and antiarteriosclerotic drugs
T46.7	Peripheral vasodilators
T46.8	Antivaricose drugs, including sclerosing agents
T46.9	Other and unspecified agents primarily affecting the cardiovascular system
T47	Poisoning by agents primarily affecting the gastrointestinal system
T47.0	Histamine H2-receptor antagonists
T47.1	Other antacids and anti-gastric-secretion drugs
T47.2	Stimulant laxatives
T47.3	Saline and osmotic laxatives
T47.4	Other laxatives
T47.5	Digestants
T47.6	Antidiarrhoeal drugs
T47.7	Emetics
T47.8	ther agents primarily affecting the gastrointestinal system
T47.9	Agent primarily affecting the gastrointestinal system, unspecified
T48	Poisoning by agents primarily acting on smooth and skeletal muscles and the respiratory system
T48.0	Oxytocic drugs
T48.1	Skeletal muscle relaxants [neuromuscular blocking agents]
T48.2	Other and unspecified agents primarily acting on muscles
T48.3	Antitussives
T48.4	Expectorants
T48.5	Anti-common-cold drugs

T48.6	Antiasthmatics, NEC
T48.7	Other and unspecified agents primarily acting on the respiratory system
T49	Poisoning by topical agents primarily affecting skin and mucous membrane and by ophthalmological, otorhinolaryngological and dental drugs
T49.0	Local antifungal, anti-infective and anti-inflammatory drugs, NEC
T49.1	Antipruritics
T49.2	Local astringents and local detergents
T49.3	Emollients, demulcents and protectants
T49.4	Keratolytics, keratoplastics and other hair treatment drugs and preparations
T49.5	Ophthalmological drugs and preparations
T49.6	Otorhinolaryngological drugs and preparations
T49.7	Dental drugs, topically applied
T49.8	Other topical agents
T49.9	Topical agent, unspecified
T50	Poisoning by diuretics and other and unspecified drugs, medicaments and biological substances
T50.0	Mineralocorticoids and their antagonists
T50.1	Loop [high-ceiling] diuretics
T50.2	Carbonic-anhydrase inhibitors, benzothiadiazides and other diuretics
T50.3	Electrolytic, caloric and water-balance agents
T50.4	Drugs affecting uric acid metabolism
T50.5	Appetite depressants
T50.6	Antidotes and chelating agents, NEC
T50.7	Analeptics and opioid receptor antagonists
T50.8	Diagnostic agents
T50.9	Other and unspecified drugs, medicaments and biological substances

International Statistical Classification of Diseases and Related Health Problems 10th Revision, ICD-10
